# Risk of other Cancers in Families with Melanoma: Novel Familial Links

**DOI:** 10.1038/srep42601

**Published:** 2017-02-15

**Authors:** Christoph Frank, Jan Sundquist, Akseli Hemminki, Kari Hemminki

**Affiliations:** 1 Division of Molecular Genetic Epidemiology, German Cancer Research Center (DKFZ), Im Neuenheimer Feld 580, D-69120, Heidelberg, Germany; 2 Center for Primary Health Care Research, Lund University, 205 02 Malmö, Sweden; 3 Stanford Prevention Research Center, Stanford University School of Medicine, Stanford, California 94305-5705, USA; 4 Cancer Gene Therapy Group, Faculty of Medicine, University of Helsinki, Finland; 5 Helsinki University Hospital Comprehensive Cancer Center, Helsinki, Finland

## Abstract

A family history of cutaneous melanoma (‘melanoma’) is a well-established risk factor for melanoma. However, less is known about the possible familial associations of melanoma with other discordant cancers. A risk for discordant cancer may provide useful information about shared genetic and environmental risk factors and it may be relevant background data in clinical genetic counseling. Using the Swedish Family-Cancer Database, we assessed the relative risk (RR) for any cancer in families with increasing numbers of first-degree relatives diagnosed with melanoma, including multiple melanoma, and in reverse order RR for melanoma in families of multiple discordant cancers. Close to 9% of melanoma was familial; among these 92% were in 2-case families and 8% in families with 3 cases or more. Cancers that were associated with melanoma, in at least two independent analyses, included breast, prostate, colorectal, skin and nervous system cancers. Other associations included cancer of unknown primary, acute myeloid leukemia/myelofibrosis and Waldenström macroglobulinemia/myeloma. Significant results, which appear biologically plausible, were also obtained for rare nasal melanoma and mesothelioma. Although small samples sizes and multiple comparisons were of concern, many of the above associations were internally consistent and provide new diverse leads for discordant familial association of melanoma.

According to the Swedish Family-Cancer Database family history of cutaneous melanoma was found for 5 to 10% of first-degree relatives (FDRs) diagnosed with this cancer, giving a familial relative risk (RR) of 2.5^1^. However, less is known about the possible familial associations of cutaneous melanoma (subsequently ‘melanoma’ if not specified) with other discordant cancers. In a systematic analysis of familial risks, between discordant sites, the highest relative risk for melanoma (1.35) was found in families of skin squamous cell carcinoma (SCC) patients. Weak associations were also found with colorectal, breast and nervous system cancers^2^. In a study from Utah, which covered three generations, two-way associations were found between melanoma and breast, female genital, lip and prostate cancers^3^. Multiple cancers in the same individual are a hallmark of a familial risk, and indeed we have found that the familial risk for melanoma was equal for persons who have 2 relatives diagnosed with a single melanoma or 1 relative with 2 primary melanomas^4^. Several discordant familial cancers, particularly pancreatic and breast cancers, were noted in persons with multiple primary melanomas^5^. The most common high-risk gene predisposing to melanoma is *CDKN2A*, which is also associated with pancreatic cancer^6^. Germline mutations in *BAP1* also predispose to cutaneous melanoma, but the mutations are relatively more important in rare cancers (uveal melanoma and mesothelioma) constituting a novel cancer syndrome^6^,^7^,^8^. Although a few other high penetrance genes are known for cutaneous melanoma their population impact is negligible because of the low number of mutation carriers.

A risk for discordant cancer may provide useful information about shared genetic and environmental risk factors and it may be relevant background data in clinical genetic counseling. In the case of melanoma, it would be pertinent to assess familial clusters, with sites which are manifested in *CDKN2A* and *BAP1* mutation carriers, or whether the risks could be extended to as yet unknown cancer sites. In this study we have applied a novel approach to search for familial associations of melanoma with other cancers using the most recent update of the Swedish Family-Cancer Database. This involves assessment of familial RRs for melanoma in families with increasing numbers of cancers X, or conversely, familial RR for cancer X in families with increasing numbers of melanomas. We additionally consider the joint occurrence of multiple primary melanomas among familial cases. Association with rare non-cutaneous melanomas was also tested.

## Results

The total number of melanomas was 79,060 and of these a total of 38,102 (including 28,495 invasive melanomas) were in the 0–80 year offspring generation for which the RRs were calculated. The case distribution by the number of FDRs diagnosed with melanoma is shown in Table 1. Only 8.57% of melanoma patients had a family history of melanoma. Among 3,267 melanoma patients with a family history, 3,007 (92.0% of melanoma with a family history) had one affected family member (2-case families), 239 (7.3%) had two affected family members (3-case families) and 21 (0.6%) had three or more affected family members (multiplex families). RRs for melanoma increased systematically from 2.42 (2-case families) to 6.49 and 8.30 (4 or more cases with melanoma).

The RR for any cancer in offspring was calculated when increasing numbers of FDRs were diagnosed with single or multiple melanomas (Table 2). The reference was families with no melanoma in FDRs; the RR in the reference families was 1.00 (not shown) and the corresponding number of cases with a negative family history of melanoma is shown in the first results column. If one family member had melanoma, increased RRs were detected for nasal (1.64; 4.89 for nasal melanoma), prostate (1.08), testicular (1.17), kidney (1.17), skin (1.46), nervous system (1.27) and thyroid gland cancers (1.28) as well as for non-Hodgkin lymphoma (1.12) and CUP of melanoma histology (2.23). If there was one FDR with multiple melanomas, significantly increased RRs for discordant associations were found for cancers of the small intestine (2.34, 3.01 for small intestinal NET), lung (1.64), breast (1.25), skin (2.00) and bone (2.60; 4.10 for osteosarcoma). Discordant cancer cases with 2 or more affected FDRs were rare. However, significantly increased risks were observed for salivary gland (3.21), colorectal (2.77), pancreatic (4.46), nasal (6.35; 21.67 for nasal melanoma), breast (1.61), cervix (2.85), prostate (1.71), skin (2.62/2.32 depending on numbers of melanomas in the family) and eye cancers (3.06; 4.15 for eye melanoma), and non-Hodgkin lymphoma (3.07), myeloma (5.02) and leukemia (1.77). A significant increasing trend, for RRs by the number of melanomas among FDR, was confirmed for colorectal, nasal, lung, breast, prostate, testicular, kidney, skin, eye, nervous system and thyroid cancers, and for non-Hodgkin lymphoma and leukemia considering 95% CIs.

In order to test the association with non-cutaneous melanomas in sites not listed in Table 2, no associations were noted for esophageal or female genital melanomas but the case numbers were few (data not shown). We similarly tested for association with NET for sites other than small intestine (stomach, appendix, rectum, nose, ovary, kidney and CUP); no associations were noted but case numbers were few (data not shown).

In view of the recently described *BAP1* associated cancer syndrome manifesting melanomas and mesotheliomas, we tested the risk of mesothelioma when cutaneous melanomas were diagnosed in FDRs. The RR was 1.55 for combined pleural and peritoneal mesothelioma (N = 51, 95% CI 1.12–2.15) when one FDR was diagnosed with melanoma and it was 2.14 (2, 0.43–10.75) when 2 FDRs were diagnosed with melanoma. The trend test was significant (p = 0.009). Neither pleural nor peritoneal mesothelioma were independently significant, but most cases (36) originated from pleural mesothelioma.

We also carried out the analysis in the reverse order (conversely), calculating RRs for melanoma when FDRs were diagnosed with any cancer (Table 3). However, as a few cancers showed multiple affected relatives only two family history categories were used (one familial cancer or two or more patients with the same familial cancer), in addition to the reference category lacking familial cancers. Significantly increased RRs were found if one FDR had nasal melanoma (2.24), or breast (1.06), prostate (1.05), skin (1.36), eye (1.35, 1.33 for eye melanoma), nervous system cancer (1.17) or CUP of melanoma histology (2.09). Individuals with 2 or more familial skin cancers showed a significant RR for melanoma of 1.78. Testing for increasing trends for RRs by the number of familial cases showed associations with colorectal, breast, prostate, skin and nervous system cancer. We separately analyzed families with 3 or more patients diagnosed with the same cancer. The RR for melanoma was significant (1.62; 27 cases, 95% CI 1.03–2.53), only for families in which 3 men were diagnosed with prostate cancer.

Based on some positive results for non-Hodgkin lymphoma and leukemia in Table 2, a more detailed analysis was conducted by considering subtypes (Table 4). RR was increased for follicular lymphoma (6.75) when at least 2 family members were diagnosed with melanoma and when at least one family member was diagnosed with multiple melanomas. With the same family history Waldenström macroglobulinemia increased to 15.45 (one case) but it was also increased (5.27) when one family member was diagnosed with multiple melanoma. Among leukemias, the risk was systematically increased for acute myeloid leukemia, reaching an RR of 7.08 in the most affected melanoma families (Table 4). Rare myelofibrosis was increased to 5.55, when 2 or more family members were diagnosed with melanoma. The analyses shown in Table 4 were also carried in reverse order but no RR was significant (data not shown).

## Discussion

The interpretation of any familial risk needs to weigh the evidence about known contributing genes and environmental factors. Data on twin or family-based heritability estimates do not provide much guidance because familial melanoma covers less than 10% of all melanoma^9^. Ultraviolet radiation is a known risk factor for melanoma and the modest correlation of melanoma risk between spouses (1.22) was suggested to be due, at least in part, to shared sun exposure among spouses^10^. The high shared risks between melanoma and skin squamous cell carcinoma shown here may be due to ultraviolet radiation, but for other cancers genetic sharing may be more important.

As to the genetic architecture of melanoma, high-risk *CDKN2A* mutations contribute to about 30% of melanomas in families of three or more affected individuals^11^. According to a recent Swedish study, *CDKN2A* positive families accounted for 11.5% (31/269) of all families, and these presented with a median of 6 melanomas compared to 2 in mutation negative families^12^. We show here that families of 3 or more diagnosed melanomas accounted for less than 8% of familial melanoma, which together with the above data suggest that *CDKN2A* mutations are likely to be, at most, a minor contributor to the present findings on discordant cancers. The case of pancreatic cancers in melanoma families is instructive in this context because *CDKN2A* is the dominant high-risk gene for both cancers^11^. The only significant RR for pancreatic cancer was the high risk of 4.46 in families with at least 2 melanomas and one with multiple melanomas; however, such families included no more than 2% (5 of 222) of the pancreatic cancer patients with a family history of melanoma.

Genome-wide association studies have been able to identify 20 genome-wide significant low-risk loci. Overall, these loci are estimated to account for 19.2% of the familial risk of melanoma^13^. Of the 20 loci, 8 were skin specific, including 5 related to pigmentation and 3 related to nevi, and an additional 4 were in regions related to telomere maintenance^13^. Considering the genetic architecture, it is conceivable that the weight of the high-risk genes can be observed in families with multiple melanoma patients, while the low-risk genes contribute predominantly to the small melanoma families. However, as many of the low-risk genes are skin specific they may not be relevant in discordant associations, except with skin cancer.

A number of novel observations emerged in this new type of analysis for cancer risk considering family histories of increasing numbers of affected family members. The focus was on discordant cancers, but for family histories only the same discordant cancers were considered. Being an exploratory study we have to be concerned about multiple comparisons. There are a limited number of earlier independent studies on the related questions and all are much smaller than the present one; thus comparison to the previous literature will provide limited reassurance. Biological plausibility and internal consistency need to be assessed. For the latter, the results are analyzed in two ways (risk of cancer by family history of melanoma, Table 2, and risk of melanoma by family history of cancer, Table 3) are largely independent (see Methods) and increased risk for both analyses should indicate a strong support for a true association. However, the two-way analysis compares cancers of different age distributions, which may reduce comparability of the two sets of results. Thus, a lack of two-way support is not strong evidence against association. A key consistency metric for true association is the trend test for RRs in families with increasing numbers of affected family members, allowing a ‘dose-response’ type of analysis. With a sufficient sample size the dose-response relationship should be consistent.

Considering internal consistency of data, i.e. evidence of dose-response or significant results on two-way analysis, skin cancer indisputably shares risk with melanoma, with ultraviolet radiation being the common risk factor while no shared contributing genes are known. Breast and prostate cancers were associated in many independent analyses with melanoma. The risk of colorectal cancer was 2.77 in multiplex melanoma families and both of the two-way trend analyses were significant. Nervous system and thyroid cancers also showed independent support for being true associations, both may also manifest *CDKN2A* mutations or deletions^6^.

Among hematologic neoplasms, associations were shown with melanoma and Waldenström macroglobulinemia, acute myeloid leukemia and myelofibrosis. Although the case numbers were small and warrant caution, there was internal consistency. Waldenström macroglobulinemia is a plasma cell disease related to myeloma and shows mutual familial risks^14^; myeloma risk was 5.02 in the most familial melanoma families, supporting the results of Waldenström macroglobulinemia (Table 2). Myelofibrosis is known to be able to progress to acute and chronic myeloid leukemia thus giving basis to the increased risk found in Table 4^15^.

It is known that ocular and cutaneous melanomas share familial risks and *BAP1* and possibly also *BRCA2* mutations may be involved^6^,^7^,^8^. However, melanomas are found, although rarely, in other locations including nasal and anogenital mucosal surfaces and the esophagus but whether they cluster with cutaneous melanoma has not been shown. Here we show that nasal melanoma was associated with cutaneous melanoma in the two-way analyses, but there was no indication for esophageal or female genital melanomas due to inconclusive data because of low case numbers. A two-way association was shown for CUP with melanoma histology. CUP is a fatal cancer where the primary tumor cannot be found and the diagnosis is based on metastases^16^. We have shown earlier familial clustering of a number of primary cancers with CUP, speculating that the associated primary cancer in the family member may point to the hidden primary in CUP^17^,^18^. The present results concur and suggest that CUP is maintaining the histology of a family member’s melanoma. We showed an association between cutaneous melanoma and mesothelioma, which could be expected as a manifestation of the novel *BAP1*-associated cancer syndrome, and would provide population-level evidence for the syndromic clustering of these cancers^6^,^7^,^8^.

The increased risk in small intestinal cancers prompted us to consider histology of the associated tumors and it was found that NETs contributed to the risk. Although the association with melanoma was surprising, the embryonic origin of these tumors may be the common denominator. Melanocytes arise in the neural crest and migrate to skin, while the neuroendocrine cells that give rise to NET are also thought to originate from this embryonic tissue^19^. However, no associations were found for NETs at other tested sites but with small case numbers.

In summary, the applied novel approach identified many cancers that were associated with melanoma, at least in two independent analyses but with few known genetic links. Breast and prostate cancers were reliably associated with melanoma but also colorectal and nervous system cancers as well as CUP, acute myeloid leukemia/myelofibrosis and Waldenström macroglobulinemia/myeloma showed fair statistical support for true associations but for which genetic bases remain unknown. Associations of melanoma with nasal melanoma and mesothelioma appear biologically plausible but more intriguing was the association with small intestinal NET. Although small samples sizes and multiple comparisons were of concern, many of the above associations were internally consistent and provide new diverse leads and incentives for genetic search beyond the present narrowly demarcated familial domain of melanoma, encompassing essentially only skin and pancreatic cancers.

## Methods

In the Swedish Family-Cancer Database 15.7 million individuals are categorized in families with cancer data from the Swedish Cancer Registry. All cancers since 1958 are registered and the latest follow-up of the database includes cancers up to and including 2012. The offspring generation is constituted of all individuals born from 1932 onwards, with parental linkage, and includes 8.5 million people; among which 427,196 cancers were diagnosed. They had reached the maximum age of 80, while the ages of their biological parents (the parental generation) were not limited.

Using the 7th revision of the International Classification of Diseases (ICD-7) the 34 most common cancers were used in the analysis; data are not shown for 4 very rare cancers. Histological type since the 1960s was recorded by pathological-anatomical coding. For only non-Hodgkin lymphomas histological type was recorded by SNOMED-coding (Systematized Nomenclature of Medicine) available from 1993. For melanoma both invasive and *in situ* diagnoses were considered. Multiple melanomas were considered only in patients who had invasive or *in situ* melanoma. The follow-up for cancer in offspring started from the beginning of 1958, the birth year, or the immigration year, whichever came latest. The follow-up was terminated when a person was diagnosed with cancer, emigrated or died, or at the end of 2012, whichever came first.

Methods of calculating familial relative risks (RRs) for individuals whose FDRs (parents and/or siblings) were diagnosed with cancer have been described elsewhere^1^. Incidence rates for persons with affected relatives were compared to rates for those whose relatives had no relevant cancer. Incidence rates were obtained by counting cases and person-years (PY) according to family history of discordant cancer:

**RR for cancer X, given family history of melanoma:** (X cases with family history of melanoma divided by PY for X with family history of melanoma) divided by (X cases without family history of melanoma divided by PY for X without family history of melanoma).

According to the converse analysis:

**RR for melanoma, given family history of X:** (Melanoma cases with family history of X divided by PY for melanoma with family history of X) divided by (melanoma cases without family history of X divided by PY for melanoma without family history of X).

Note that none of the terms are identical in these two-way analyses, implying that for offspring-parent pairs the results are independent but for siblings the pairs of cases are the same.

RRs were stratified for sex, age group, calendar period, residential area and socioeconomic status to account for potential confounders. These variables were used as covariates in a Poisson regression model to get adjusted RRs and corresponding confidence intervals (CIs). Trend tests were performed by modeling the number of familial cancers as a continuous covariate.

### Ethical statement

The study was approved by the Ethical Committee of Lund University and the study was conducted in accordance with the approved guidelines.

## Additional Information

**How to cite this article**: Frank, C. *et al*. Risk of other Cancers in Families with Melanoma: Novel Familial Links. *Sci. Rep.* **7**, 42601; doi: 10.1038/srep42601 (2017).

**Publisher's note:** Springer Nature remains neutral with regard to jurisdictional claims in published maps and institutional affiliations.

## Figures and Tables

**Table 1:** Distribution of melanoma cases by the number of melanoma patients in family members and relative risk^a^.

Affected relatives	Frequency	RR	95% CI	% in melanoma (% familial melanoma)
0	34835	1.00	—	91.43 (−)
1	3007	2.42	2.30–2.54	7.89 (92.0)
2	239	6.49	5.44–7.76	0.63 (7.3)
3	21	8.30	6.65–10.01	0.06 (0.6)

^a^Only patients with first invasive or *in situ* melanoma were considered.

**Table 2:** Risk of cancer in families with increasing numbers of melanoma patients and multiple melanomas.

Cancer site	Cases negative family history	1 FDR had melanoma			1 FDR had multiple (≥2) melanomas			≥2 FDRs had melanoma			≥2 FDRs had melanoma,≥1 FDR had multiple melanomas			P-trend test for RR
		Cases	RR	95% CI	Cases	RR	95% CI	Cases	RR	95% CI	Cases	RR	95% CI	p-value
Upper aerodigestive tract	8416	252	0.92	(0.74–1.14)	22	1.33	(0.65–2.72)	5	0.67	(0.15–2.99)	2	2.58	(0.10–12.23)	0.60
Salivary glands	1091	36	1.01	(0.72–1.41)	3	1.37	(0.44–4.29)	3	**3.21**	(1.02–10.07)	0			0.31
Stomach	5244	160	0.99	(0.81–1.21)	6	0.65	(0.24–1.79)	9	2.09	(0.92–4.78)	0			0.73
Small intestine	1757	55	0.96	(0.74–1.24)	8	**2.34**	(1.19–4.59)	1	0.64	(0.10–4.31)	0			0.61
NET	850	30	1.08	(0.74–1.57)	5	**3.01**	(1.21–7.50)	1	1.33	(0.17–10.20)	0			
Colorectum	34098	1137	1.05	(0.98–1.13)	70	1.10	(0.84–1.44)	36	1.23	(0.84–1.79)	19	**2.63**	(1.61–4.60)	**0.0052**
Liver	5692	184	1.09	(0.93–1.27)	10	1.04	(0.53–2.04)	4	0.91	(0.31–2.61)	3	2.68	(0.83–9.88)	0.22
Pancreas	6369	201	1.05	(0.84–1.30)	8	0.75	(0.26–2.18)	8	1.58	(0.54–4.61)	5	**4.32**	(1.11–17.11)	0.49
Nose	554	29	**1.64**	(1.09–2.45)	2	1.87	(0.42–8.43)	3	**6.35**	(1.86–21.70)	0			**0.0015**
Nasal melanoma	51	8	**4.89**	(2.13–11.20)	0			1	**21.67**	(2.40–195)	0			**0.0002**
Lung	23546	731	1.02	(0.94–1.11)	67	**1.64**	(1.25–2.16)	24	1.25	(0.79–1.97)	9	2.03	(0.97–4.34)	**0.0138**
Breast	80034	2861	1.04	(1.00–1.08)	215	**1.25**	(1.08–1.45)	126	1.61	(1.33–1.96)	18	0.99	(0.58–1.61)	**<0.0001**
Cervix	8621	306	1.06	(0.93–1.20)	13	0.71	(0.38–1.32)	5	0.67	(0.25–1.80)	5	**2.75**	(1.02–7.68)	0.70
Endometrium	10877	348	0.99	(0.82–1.20)	18	0.86	(0.38–1.95)	5	0.52	(0.11–2.44)	6	2.30	(0.53–9.83)	0.84
Ovary	9595	339	1.07	(0.96–1.19)	26	1.35	(0.93–1.96)	8	0.94	(0.48–1.84)	2	0.91	(0.20–3.76)	0.12
Prostate	59570	2074	**1.08**	(1.03–1.13)	132	1.17	(0.99–1.40)	66	1.21	(0.94–1.54)	22	**1.71**	(1.11–2.64)	**<0.0001**
Testis	7222	266	**1.17**	(1.01–1.35)	14	0.94	(0.50–1.75)	9	1.68	(0.77–3.65)	2	1.60	(0.30–8.65)	**0.0314**
Kidney	9088	325	**1.17**	(1.04–1.31)	16	0.97	(0.58–1.63)	11	1.48	(0.80–2.77)	0			**0.0127**
Urinary bladder	13432	443	1.04	(0.94–1.16)	33	1.32	(0.91–1.92)	12	1.02	(0.55–1.91)	5	1.63	(0.61–4.63)	0.14
Skin, squamous cell	23648	1130	**1.46**	(1.37–1.56)	94	**2.00**	(1.60–2.50)	57	**2.62**	(1.97–3.49)	13	**2.39**	(1.29–4.38)	**<0.0001**
Eye	1779	66	1.26	(0.98–1.61)	4	1.24	(0.47–3.30)	4	**3.06**	(1.15–8.13)	0			**0.0197**
Eye melanoma	828	31	1.14	(0.83–1.57)	3	1.83	(0.67–4.97)	3	**4.15**	(1.53–11.29)	0			**0.0357**
Nervous system	21364	827	**1.27**	(1.16–1.39)	51	1.27	(0.89–1.80)	23	1.39	(0.82–2.36)	5	1.31	(0.40–4.09)	**<0.0001**
Thyroid gland	5344	224	**1.28**	(1.10–1.48)	10	0.91	(0.46–1.81)	3	0.67	(0.19–2.33)	1	**9.07**	(1.31–37)	**0.0454**
Endocrine glands	9302	328	1.07	(0.93–1.23)	22	1.17	(0.69–1.98)	12	1.47	(0.72–3.01)	5	3.04	(0.83–7.92)	0.14
Bone	1833	64	1.17	(0.85–1.62)	9	**2.60**	(1.11–6.09)	1	0.78	(0.06–9.95)	1	3.44	(0.24–40)	0.06
Osteosarcoma	676	21	1.10	(0.71–1.70)	5	**4.10**	(1.70–9.92)	0			0			0.12
Connective tissue	3633	129	1.15	(0.93–1.41)	7	1.01	(0.42–2.42)	1	0.35	(0.04–3.54)	0			0.41
Non-Hodgkin lymphoma	13803	485	**1.12**	(1.02–1.23)	32	1.22	(0.85–1.75)	13	1.13	(0.64–1.98)	8	2.88	(1.32–6.19)	**0.0021**
Hodgkin lymphoma	4277	145	1.10	(0.88–1.38)	13	1.54	(0.74–3.23)	2	0.64	(0.10–4.16)	0			0.26
Myeloma	3940	130	1.02	(0.85–1.24)	10	1.33	(0.68–2.62)	3	0.86	(0.25–2.96)	4	**4.77**	(1.65–14.51)	0.21
Leukemia	14141	439	1.08	(0.96–1.20)	32	1.29	(0.86–1.94)	18	**1.77**	(1.03–3.04)	4	1.64	(0.52–5.41)	**0.0169**
CUP	8827	311	1.17	(0.96–1.43)	23	1.54	(0.75–3.16)	10	1.45	(0.49–4.33)	0			0.06
CUP melanoma	683	50	**2.23**	(1.45–3.44)	4	2.98	(0.68–13.03)	1	1.68	(0.09–31.84)	0			**0.0004**

**Table 3:** Risk of melanoma in families of increasing numbers of patients with discordant concordant cancers.

Cancer site family member	Cases negative family history	1 discordant case			≥2 discordant cases^a^			Trend test for RR
		Cases	RR	95% CI	Cases	RR	95% CI	p-value
Upper aerodigestive tract	37529	568	0.96	(0.87–1.06)	5	0.96	(0.35–2.63)	0.40
Salivary glands	38022	80	1.23	(0.92–1.63)	0			
Stomach	37189	904	0.95	(0.88–1.03)	9	0.71	(0.32–1.56)	0.15
Small intestine	37964	137	1.14	(0.94–1.39)	1	2.05	(0.20–21.05)	0.18
NET	38032	70	1.10	(0.81–1.49)	0			
Colorectum	34967	2980	1.05	(1.00–1.11)	155	1.24	(0.99–1.56)	**0.0150**
Liver	37452	643	0.95	(0.86–1.05)	7	1.15	(0.44–3.04)	0.38
Pancreas	37435	661	0.96	(0.87–1.06)	6	0.83	(0.29–2.32)	0.33
Nose	38060	42	0.93	(0.58–1.49)	0			
Nasal melanoma	38090	12	**2.64**	(1.23–5.68)	0			
Lung	36201	1834	0.97	(0.92–1.04)	67	1.08	(0.79–1.48)	0.56
Breast	34249	3660	**1.06**	(1.02–1.10)	193	1.10	(0.93–1.31)	**0.0028**
Cervix	37644	456	0.91	(0.81–1.01)	2	0.78	(0.15–4.04)	0.07
Endometrium	37354	744	1.00	(0.92–1.08)	4	0.53	(0.18–1.57)	0.75
Ovary	37445	650	1.07	(0.98–1.17)	7	1.31	(0.56–3.04)	0.13
Prostate	33840	4029	**1.05**	(1.01–1.10)	233	1.09	(0.93–1.28)	**0.0081**
Testis	37987	114	1.10	(0.88–1.37)	1	1.54	(0.14–16.64)	0.40
Kidney	37277	815	1.07	(0.98–1.16)	10	1.33	(0.62–2.86)	0.12
Urinary bladder	36833	1245	1.00	(0.93–1.08)	24	1.12	(0.67–1.88)	0.84
Skin, squamous cell	35298	2681	**1.36**	(1.30–1.43)	123	**1.78**	(1.42–2.22)	**<0.0001**
Eye	37992	109	**1.35**	(1.05–1.74)	1	6.65	(0.48–91.93)	0.0543
Eye melanoma	38019	83	**1.33**	(1.01–1.74)	0			
Nervous system	37138	949	**1.17**	(1.08–1.26)	15	1.40	(0.78–2.51)	**<0.0001**
Thyroid gland	37863	238	1.09	(0.93–1.27)	1	0.74	(0.07–8.07)	0.32
Endocrine glands	37580	515	1.10	(0.99–1.22)	7	1.72	(0.70–4.22)	0.07
Bone	38061	41	0.87	(0.60–1.26)	0			
Osteosarcoma	38096	6	0.56	(0.18–1.75)	0			
Connective tissue	37892	210	1.19	(0.97–1.47)	0			
Non-Hodgkin lymphoma	37246	851	1.04	(0.95–1.14)	5	0.50	(0.15–1.61)	0.60
Hodgkin lymphoma	37977	125	0.96	(0.77–1.21)	0			
Myeloma	37700	399	1.05	(0.93–1.19)	3	1.18	(0.28–4.98)	0.45
Leukemia	37281	810	1.08	(0.98–1.18)	11	1.21	(0.56–2.66)	0.10
CUP	37214	882	1.04	(0.96–1.13)	6	0.76	(0.29–1.97)	0.41
CUP melanoma	38042	60	**2.09**	(1.44–3.05)	0			

^a^Family members had the same discordant cancers, e.g., 2 or more lung cancers.

**Table 4:** Risk for specific subtypes of non-Hodgkin lymphoma and leukemia in families with increasing number of melanoma patients and multiple melanomas.

Cancer site	Cases negative family history	1 FDR had melanoma			1 FDR had multiple (≥2) melanomas			≥2 FDRs had melanoma			≥2 FDRs had melanoma, ≥1 FDR had multiple melanomas			Trend test for RR
		Cases	RR	95% CI	Cases	RR	95% CI	Cases	RR	95% CI	Cases	RR	95% CI	p-value
Diffuse large cell	2911	93	1.02	(0.82–1.26)	8	1.47	(0.72–2.99)	1	0.41	(0.06–3.09)	0			0.84
Follicular	2148	74	1.03	(0.80–1.33)	3	0.69	(0.20–2.38)	4	2.07	(0.71–6.04)	3	**6.75**	(1.96–23.25)	0.16
Waldenström	294	8	0.83	(0.42–1.64)	3	5.27	(1.75–15.91)	1	3.65	(0.54–24.55)	1	**15.45**	(2.30–104)	0.11
Hairy cell	335	16	1.39	(0.79–2.44)	0			1	3.16	(0.35–28.48)	0			0.17
Acute lymphoid leukemia	3257	73	1.08	(0.87–1.33)	7	1.68	(0.84–3.34)	1	0.83	(0.13–5.15)	0			0.29
Chronic lymphoid leukemia	3075	116	1.17	(0.97–1.42)	7	1.21	(0.57–2.56)	2	0.73	(0.18–3.01)	1	1.63	(0.22–11.92)	0.16
Acute myeloid leukemia	2548	75	0.99	(0.75–1.30)	13	**2.85**	(1.48–5.48)	6	**3.19**	(1.22–8.35)	3	**7.08**	(1.81–27.61)	**0.0239**
Chronic myeloid leukemia	1273	37	0.90	(0.61–1.31)	3	1.17	(0.31–4.38)	2	1.88	(0.37–9.50)	0			0.96
Polycythemia vera	1075	45	1.26	(0.97–1.65)	0			1	1.02	(0.18–5.87)	0			0.13
Myelofibrosis	829	27	1.01	(0.68–1.52)	1	0.62	(0.08–4.91)	4	**5.55**	(1.97–15.59)	0			0.18
